# Perspectives on Nanomaterials and Nanotechnology for Sustainable Bioenergy Generation

**DOI:** 10.3390/ma15217769

**Published:** 2022-11-03

**Authors:** Kalaimani Markandan, Wai Siong Chai

**Affiliations:** 1 Department of Chemical & Petroleum Engineering, Faculty of Engineering, Technology and Built Environment, UCSI University, Kuala Lumpur 56000, Malaysia; 2 Department of Mechanical and Electro-Mechanical Engineering, National Sun Yat-sen University, Kaohsiung 80424, Taiwan

**Keywords:** nanomaterials, bioenergy generation, enzyme immobilization, biohydrogen, biogas, nanotechnology

## Abstract

The issue of global warming calls for a greener energy production approach. To this end, bioenergy has significant greenhouse gas mitigation potential, since it makes use of biological products/wastes and can efficiently counter carbon dioxide emission. However, technologies for biomass processing remain limited due to the structure of biomass and difficulties such as high processing cost, development of harmful inhibitors and detoxification of produced inhibitors that hinder widespread usage. Additionally, cellulose pre-treatment is often required to be amenable for an enzymatic hydrolysis process. Nanotechnology (usage of nanomaterials, in this case) has been employed in recent years to improve bioenergy generation, especially in terms of catalyst and feedstock modification. This review starts with introducing the potential nanomaterials in bioenergy generation such as carbon nanotubes, metal oxides, silica and other novel materials. The role of nanotechnology to assist in bioenergy generation is discussed, particularly from the aspects of enzyme immobilization, biogas production and biohydrogen production. Future applications using nanotechnology to assist in bioenergy generation are also prospected.

## 1. Introduction

Research on bioenergy generation has been growing at a relentless pace owing to the concerns that arose from dwindling fossil fuel reserves and the environmental pollution associated with the exploitation of these resources. Studies have reported that emission of greenhouse gases such as carbon dioxide (CO_2_) from the burning of fossil fuels has led to climate change issues [1,2]. Since the transportation sector accounts to 60% of the estimated oil demand for the year 2030 (116 million barrels per day), it is essential to replace fossil-fuel-based energy sources in vehicles with renewable energy sources [3,4,5]. In an attempt to gradually replace petroleum and coal resources, various renewable energy sources such as solar, wind, hydrothermal and biomass have been explored to date, with an increased interest in the latter, since lignocellulosic biomass is available abundantly from agriculture and forestry, with an estimated annual production of 2 × 10^11^ tonnes [6]. It was also reported that in 2011, 38 million tonnes of biomass were used for biofuels in the EU, out of 1.2 billion tonnes of biomass that were generated from various crops [7].

**Figure 1:**
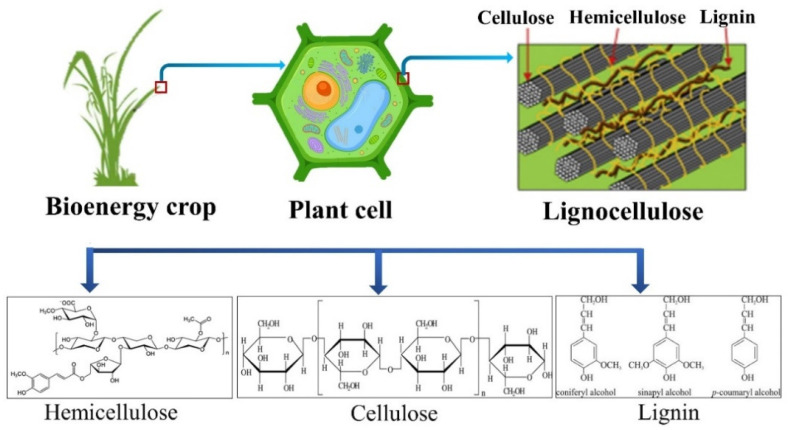
Lignocellulosic biomass as bioenergy crop; plant cell wall is made of lignocellulose containing carbohydrate polymers (cellulose, hemicellulose and aromatic polymer lignin). The cellulose and hemicellulose accommodate 6 and 5 carbon sugars, which are rigidly bounded with the lignin.

Despite the availability of lignocellulosic biomass in abundance, the technologies for biomass processing remain limited. For instance, the biomasses are recalcitrant in nature due to their cellulose crystallinity and non-reactive lignin, thus requiring pre-treatment techniques to allow the cellulose to be amenable for an enzymatic hydrolysis process [8]. This is required for the extraction of fermentable sugar for subsequent biofuel generation processes [9,10]. Figure 1 shows the cellulose and hemicellulose that accommodate 6 and 5 carbon sugars rigidly bounded with the lignin, where cellulose forms structure of the cell walls, while hemicellulose assists in the cross-linking between the non-cellulosic and cellulosic polymer via covalent bonding [11]. It is noteworthy that biomass resources currently deployed as feedstock in human or non-human food chains should not be used for chemical processing to ensure sustainability. For instance, wheat straw is a common fodder for raising animals and therefore is available in short supply due to increase in meat consumption; wheat straw should not be considered as a viable option for bioenergy generation [12].

Generally, biochemical or thermochemical routes can be employed for the conversion of lignocellulosic raw materials into bioenergy. The biochemical route uses microorganisms and various enzymes to convert components of the feedstock into sugars, followed by fermentation to produce ethanol [13]. Since the biochemical route uses various enzymes to convert feedstock to biofuel, the efficacy of the enzyme is important to improving the conversion process. On the other hand, the thermochemical route involves gasification technologies to produce simple sugars that are fermentable for biofuel production. In a study by Daystar et al. [14], it was highlighted that thermochemical conversion can use a wider range of feedstock compared to biochemical conversion and produces considerably higher alcohol yields. Additionally, unlike the biochemical process, the thermochemical process is not affected by the lignin in the biomass, although presence of moisture content can affect alcohol yield as well emissions in the thermochemical process. A general finding from most studies indicates that high cost and limited availability of existing infrastructure have posed limitations on obtaining high quality and yield of bioenergy.

However, with the emergence of nanotechnology, the limitations can be addressed efficiently since nanotechnology involves nanomaterials, which exhibit unprecedented characteristics and properties, all of which are highly useful in areas of bioenergy generation. For example, nanotechnology has proven to be advantageous in the bioenergy field for various applications such as feedstock modification and development of efficient catalysts for the hydrolysis of biomass to produce biofuels such as ethanol and biogas or for the catalysis of biodiesel production from oils and fats [15,16,17,18,19,20]. Within this context, various nanoparticles such as magnetic nanoparticles (MNPs), carbon nanotubes (CNTs), porous metal oxides, mesoporous silica and metallic organic frameworks (MOFs) have been commonly used to replace enzymes or immobilize them, which results in an efficient catalysis process for the production of bioenergy.

Whilst much of the emphasis has been intensifying on experimental studies of improving bioenergy yield of lignocellulosic biomass with the aid of nanoparticles, comprehensive review on how nanotechnology and nanomaterials affect bioenergy production is still limited. Herein, the authors make an attempt to gather information from the early to the most recent developments regarding the various nanomaterials and their role in bioenergy generation. More specifically, the paper is aimed at discussing three principle topics in depth: (i) the various nanomaterials in bioenergy generation (MNPs, CNTs, porous metal oxides, mesoporous silica and MOFs), (ii) the role of nanoparticles in bioenergy generation and (iii) the role and importance of nanotechnology in bioenergy generation. Lastly, we highlight the challenges of and prospects for future progress in bioenergy generation from lignocellulosic biomasses utilizing nanomaterials.

## 2. Potential Nanomaterials in Bioenergy Generation

The International Standardization Organization (ISO) has classified nanomaterials as any material with either its external, internal or surface structure having a dimensions in the nanoscale range of 1–100 nm [21]. In most cases, nanostructured materials are classified based on their dimensionality, such as zero-dimensional (e.g., graphene/carbon/inorganic quantum dots, fullerenes, magnetic nanoparticles), one-dimensional (e.g., nanotubes, nanofibers and nanowires), two-dimensional (e.g., nanosheets, nanoflakes of: graphene, boron nitride, MXenes, transition metal dichalcogenide) or three-dimensional (nanophase materials composed of equiaxed nanometer-sized grains) materials. The physical and chemical properties of nanoparticles (NPs) vary significantly from their macro counterparts, which have been widely exploited for use in bioenergy generation. For instance, the faster reaction rate (high catalytic activity) of NPs owing to the relatively fine particle size of NPs (i.e., large surface-area-to-volume ratio, which increases the number of active sites for various reactions to occur) is highly advantageous in the bioenergy generation process. Furthermore, other characteristics of NPs such as crystallinity, durability, stability, adsorption capability, and efficient storage have been reported to be highly desirable in the bioenergy generation field to enhance the productivity, hydrolysis and stability of cellulase enzymes [22,23].

NPs can be broadly classified as organic or inorganic nanoparticles. Examples of organic NPs are liposomes, polymersomes, polymer constructs and micelles, all of which have found great usage in imaging or drug and gene delivery techniques. On the other hand, inorganic NPs such as gold, quantum dots, carbon nanotubes and magnetic nanoparticles have drawn significant research and commercial interest in bioenergy production owing to their excellent physical properties (optical, magnetic) as well as their chemical properties (inertness, stability and ease of functionalization). Hybrid NPs, which combine both organic and inorganic NPs, exhibit the properties of both parent NPs and have shown improved performances (better catalyst recovery, higher selectivity) over the parent NPs in certain cases. Recently, single-atom catalysts (SACs) possessing single-atom centers decorated on support have drawn significant research interest among the scientific community due to biomass conversion attributed to its unique geometric configuration, electronic properties and ensemble effect [24]. In comparison to a traditional catalytic system, the SACs combine advantages of both homogenous and heterogenous catalysts by showcasing enhanced performance, increased thermal and chemical stability (i.e., due to stabilization of metal center on support) and easy recoverability. In fact, it has been reported that SACs displayed excellent catalytic activity and selectivity, i.e., 95% in most cases [25]. The following section will review the various NPs that have been used in bioenergy production.

### 2.1. Magnetic Nanoparticles (MNPs)

**Figure 2:**
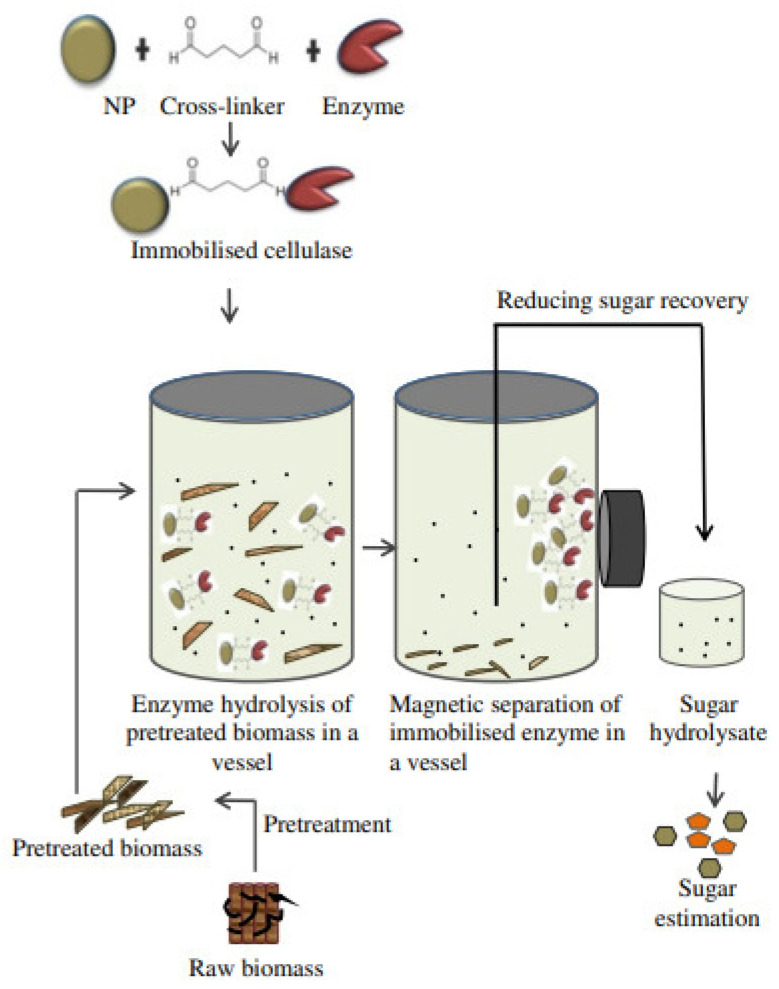
Schematic illustration describing the use of magnetic nanoparticles (MNPs) immobilized cellulase enzyme in hydrolysis process of lignocellulosic biomass for biofuel production. Reproduced from [32] with permission from Springer Nature.

The magnetic properties of MNPs are a result of the intrinsic properties of the particles as well as the interaction between the particles. MNPs perform optimally when the particle size is below the critical value of 10–20 nm, since at this size range, NPs behave as a single magnetic domain, and with application of a magnetic field, they behave as giant paramagnetic atom [3,26]. Some advantages of MNPs include their high surface-area-to-volume ratio, excellent quantum properties and small size, which enables them to carry other compounds. Examples of materials that have been used to build MNPs include cobalt, iron, nickel and platinum [27,28]. One of the major advantages of MNPs that supersedes their counter NPs is the ability to be coated and used as catalyst through immobilization, which can later be easily removed (recoverability) via application of a suitable magnetic field [29,30]. However, one major limitation of MNPs is their intrinsic instability over a large duration of time, during which the small particles form agglomerates to reduce the energy associated with the high surface-area-to-volume ratio. Additionally, since the MNPs are chemically very active, they can easily lose their magnetic properties by oxidation and dispersion. As such, strategies such as coating or modification with surfactants or inorganic layers of silica and carbon can be essential to protect the MNPs against chemical destruction during and after the synthesis stages [31]. The functionalized MNPs can be useful in bioenergy production, since they will be highly stable (i.e., highly dispersible), reactive and easily separable at later stages. A schematic illustration describing the use of MNPs immobilized cellulase enzyme in hydrolysis of lignocellulosic biomass for the production of biofuel is shown in Figure 2.

### 2.2. Carbon Nanotubes (CNTs)

CNTs consist of graphite sheets rolled up into a cylindrical shape with a nanometric diameter, and they have excellent biocompatibility and mechanical strength. Additionally, CNTs have a large surface area, which enables a high capacity to load enzymes and low diffusion resistance [33]. However, the biocompatibility of CNT composite is affected by CNT amount and length, as well as the blended substance, which might render it carcinogenic [34]. All the excellent properties of CNTs are appealing for use in enzyme immobilization. The ability of CNTs to attach themselves to the rooted sites of enzymes for direct electron transfer and the 3D electro-active area of CNTs, which increases enzyme concentration and other redox compounds on its surface, have made CNTs a very important material in biofuel applications [35,36]. Specifically, multi-walled CNTs (MWCNTs) perform comparatively better than single-walled CNTs (SWCNTs) since the immobilization of enzymes is highly compatible with the structural arrangement of MWCNTs, which further enhances the catalytic activities of immobilized enzymes. For example, Ahmad et al. [37] and Mubarak et al. [38] reported that MWCNTs outperformed hydrolysis of cellulose from *Aspergillus niger*, where an efficiency of 85% to 97% was attained, while retaining recyclability by 52% to 75% after six cycles of a hydrolysis process. Surface functionalization of CNTs has also been reported to further enhance the catalytic activity of the immobilized enzymes. For example, in a study by Pavlidis et al., the immobilization of lipase on the functionalized MWCNTs supports using the cross-liner glutaraldehyde enabled the biomaterial to retain >55% of its activity after 6 months at 4 °C and 25% of their initial activity after 30 days of incubation in hexane at 60 °C [39].

### 2.3. Porous Metal Oxides

Mesoporosity in metal oxides can be achieved by using various templates such as block copolymers [40], amines [41] or surfactants [42]. Additionally, some studies have also reported that mesopores can be introduced on the surface of metal oxide particles using soft templates such as aspartic acid and salicylic acid to convert glucose and fructose to 5- hydroxymethylfurfural (HMF). For instance, in a study by Dutta et al. [43], using sodium salicylate as a template, direct conversion of carbohydrates into HMF was investigated over a self-assembled mesoporous TiO_2_ nanoparticles (NPs) catalyst. It was reported that the high surface area for the self-assembled mesoporous TiO_2_ nanomaterials along with the Lewis acidity enabled high catalytic activity, which was able to produce 34.3% and 54.1% HMF in water and dimethyl sulfoxide (DMSO), respectively. Similarly, in another study by De at al. [44], the biopolymer sodium alginate templated porous TiO_2_ nanocatalyst with large pores in nanoscale dimension at the material surface assisted in the high catalytic activity, as under hydrothermal conditions, the material was able to catalyze the transformation of unutilized sugar derivates (i.e., D-mannose, D-galactose and lactose) to HMF under microwave irradiation at 140 °C, producing up to 44% yield. On the other hand, the TiO_2_-H was capable of retaining its catalytic activity for four cycles, suggesting its high importance in biomass conversion. Other studies have used reduced copper porous metal oxides for the supercritical methanol depolymerization and hydrodeoxygenation of biomass [45], mesoporous Al_2_O_3_ for the dehydration reaction for conversion of glucose to HMF [46] and highly ordered mesoporous sulfated zirconium for biodiesel production from oleic acid and jatropha oil [47]. Additionally, Sn or Zr metals have been the more preferred option compared to Ti metals, since they have low electronegativity, and hence the metal protons can be released easily; thus, SO_4_^2−^/SnO_2_ or SO_4_^2−^/ZrO_2_ show stronger acidity compared to SO_4_^2−^/TiO_2_, while the low surface area of the sulfated nonporous oxide also restricts reactants from accessing the active acid sites [48].

### 2.4. Mesoporous Silica

The properties of mesoporous silica, such as large surface area and pore volume with tunable pore diameter, simple surface functionalization, hydrothermal stability and biocompatibility, have been reported to be advantageous in immobilization of a range of enzymes [49,50]. For instance, the mesoporous silica material SBA-15 has been commonly used in comparison to MCM-41, owing to its large pore diameter and its mechanical and hydrothermal stability. In particular, the pores attached to organic groups such as -SO_3_H and -COOH with uniformly distributed pore size are highly essential within the class of mesoporous materials, since they play an important role in the field of heterogenous catalysis. For example, the silica-based materials, which have a high concentration of silanol groups, are important in covalent immobilization of active sites, which also allows for additional organic functionality to control the surface hydrophobicity, all of which are characteristics highly desirable to enhance aspects of catalytic performance such as durability, activity and selectivity in acid-catalyzed chemical reactions [51].

**Figure 3:**
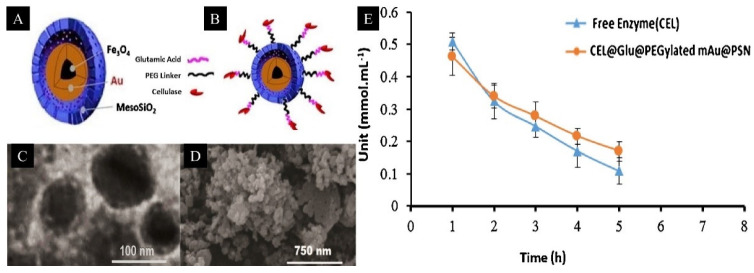
(**A**,**B**) Schematic illustration of immobilized enzyme; cellulase (CEL) immobilized glutamic-acid-functionalized magnetic gold mesoporous silica nanoparticle core shells (CEL@Glu@PEGylated mAu@PSN). (**C**,**D**) TEM/SEM images of Glu@PEGylated mAu@PSNs. (**E**) Change in enzymatic activity of free and immobilized cellulase with time at pH = 4.8 and 50 °C. Reproduced from [53] with permission from Elsevier.

In many studies, active sites of functionalized mesoporous silica have been reported to be appealing for biomass conversion. For example, in a study by Dufaud and Davis, it was shown that mesoporous silica (SBA-15) with active sites such as sulfonated acid using organic–inorganic hybrid silane showed enhanced catalytic activity and selectivity [52]. In another study by Poorakbar et al. [53], magnetic gold mesoporous silica nanoparticle core shells were fabricated as a support for cellulase immobilization via covalent bonding, and it was reported that the immobilized enzyme maintained 58% of its initial catalytic activity even after 9 h. Additionally, the support for cellulase immobilization exhibited enhanced thermal stability, applicable for a broader temperature and pH range, while showcasing its capability for long-term storage as well as easy separability using an external magnet (Figure 3). A study by Lee et al. [54] used Fe_3_O_4_ encapsulated mesoporous silica nanoparticles for cellulosic biomass conversion, and it was reported that a maximum fructose yield of 51% was achievable under optimized reaction temperature (50 °C), time (24 h) and pH values (phosphate buffer with pH 4.8). Authors have highlighted the effectiveness of Fe_3_O_4_ -enzyme immobilized mesoporous silica nanoparticles for the deconstruction of glycoside bonds of cellulose at a pH of 4.8 and the possibility of integrating enzymatic and chemocatalytic biomass processing for various catalytic applications. Other studies have reported using molybdenum supported on KIT-5 mesoporous silica catalyst for the production of biofuels and value-added chemicals (furans and phenols) from biomass through catalytic fast pyrolysis of pine [55], organically modified biogenic mesoporous silica nanoparticles (RKIT-6) for xylanase immobilization for lignocellulosic biomass hydrolysis [56] and nickel nanoparticles supported on 2D (COK-12) and 3D (KIT-6 and SBA-16) mesoporous silicas for the hydrocyclization of biomass-derived levulinic acid [57].

### 2.5. Metal Organic Frameworks (MOF)

**Figure 4:**
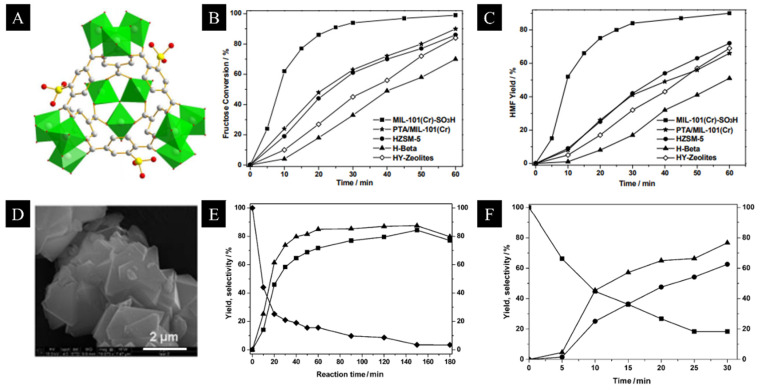
(**A**–**C**) Structure of sulfonic-acid-functionalized MOF as catalyst: MIL-101(Cr)-SO_3_H for conversion of fructose into HMF, the fructose conversion over time and HMF yield against time. (**D**) SEM image of catalyst prepared by adsorbing phosphotungstic acid (PTA) in MIL-101 MOF, (**E**) yield and selectivity over time for fructose dehydration using (PTA)-MIL-101 in 1-ethyl-3-methlyimidazolium chloride at 80 °C; square, diamond and triangular points indicate fructose retention, HMF yield and HMF selectivity, respectively (**F**) yield and selectivity over time for fructose dehydration using (PTA)-MIL-101 in DMSO at 130;square, circular and triangular points indicate fructose retention, HMF yield and HMF selectivity, respectively. Reproduced from [58,59] with permission from John Wiley and Sons and the Royal Society of Chemistry.

Metal organic frameworks (MOF) are highly useful in heterogenous catalysts, since they have high surface area with modifiable metal–organic frameworks. Researchers have utilized the merits of MOF to develop MOF-derived solid acid as a heterogenous catalyst for the biotransformation of carbohydrates to HMF, and it has been reported that the high porosity and ordered structure of the catalyst enables more substrate transfer within the MOF catalyst. For instance, the first example of MOF application in carbohydrate dehydration was reported in a study by Zhang et al. [58] using MIL-101, a chromium-based MOF that is well known for its large pore size, large surface area and good stability. Phosphotungstic acid (H_3_PW_12_O_40_, PTA) encapsulated in MIL-101 was used as the potential catalyst for the selective dehydration of fructose and glucose to HMF, where under optimal conditions HMF yield of 79% was attained from fructose after 2.5 h (Figure 4). In another study by Chen at al., a series of sulfonic-acid-functionalized MOF, MIL-101 (Cr)-SO_3_H, was used as the catalyst for the conversion of fructose into HMF, and it was reported that an HMF yield of 90% was obtained with full fructose conversion at 120 °C for 60 min in DMSO. MIL-101(Cr)-SO_3_H behaved as the heterogenous catalyst and has easy recoverability and reusability, all of which highlights the good prospects of MOF-derived solid acid catalyst for biomass carbohydrate valorization [59].

Cellulose and cellobiose were also selectively converted to sorbitol over phosphotungstic acid (PTA)-MOF hybrid supported ruthenium catalysts Ru-PTA/MIL-100(Cr) under aqueous hydrogenation conditions, where under optimal conditions, 63.2% yield in hexitol with sorbitol selectivity of 57.9% at complete conversion of cellulose and 97.1% yield in hexitol and 95.1% selectivity of sorbitol at complete conversion of cellobiose were obtained [60]. This study provided a breakthrough in perspectives for the rational design of acid–metal bifunctional catalysts for efficient biomass conversion. Other studies have reported that post-synthetic modification of the MOF functional group, which contains an organic ligand component, is more advantageous than conventional carbon materials or inorganic solids [61,62].

### 2.6. Zeolites

**Figure 5:**
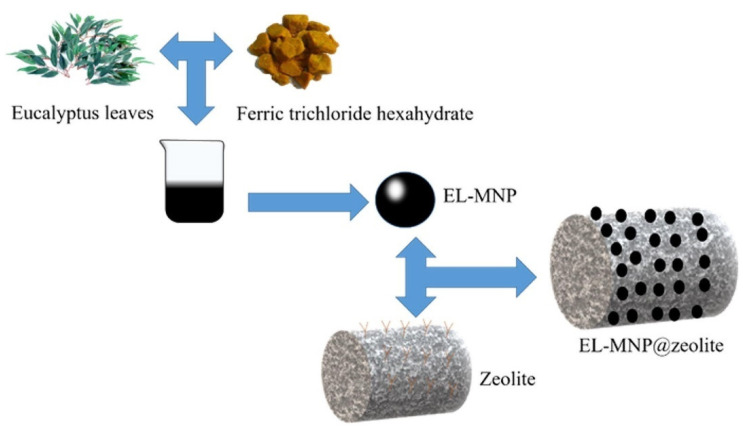
Schematic diagram describing the synthesis of iron-oxide-nanoparticle–zeolite-based hybrid MNP, where ferric trichloride was initially added to eucalyptus leaf extract and stirred for an hour at 70 °C before being filtered. In the second stage, zeolite was added to the eucalyptus–MNP solution, and in the final stage, the eucalyptus–MNP–zeolite solid was filtered and washed with anhydrous ethanol and deionized water before drying in vacuum oven at 45 °C for 12 h. Reproduced from [68] with permission from Elsevier.

Zeolites have strong Brønsted and Lewis acid sites with well-arranged micropores and excellent thermal stability owing to their crystalline inorganic framework and have been commonly used as a principle heterogenous catalyst in petrochemical and fine industrial plants. However, it is noteworthy that limitations of zeolites such as sensitivity to hot water have limited their usage in aqueous phase processes such as biomass conversion. A schematic diagram illustrating the synthesis of an iron-oxide-nanoparticle–zeolite-based hybrid MNP is shown in Figure 5.

Among the numerous zeolites, ZSM-5 based catalysts have been extensively researched, and in a comprehensive review written by Stocker et al., it was reported that the active acidic sites of zeolite (i.e., H-ZSM) that behaves as carbonium ion can be effective in the pyrolysis process of wood biomass [63]. The acidity and porosity of ZSM-5 based catalysts can be tuned by altering the content of Si or Al in zeolite. For example, in a study by Srinivasan et al., SiO_2_/Al_2_O_3_ ratios of zeolite (ZSM-5) catalyst in aromatic hydrocarbon production through catalytic pyrolysis of cellulose were investigated, and it was shown that acidity of catalysts plays an essential role in eliminating anhydro sugars and other oxygenated compounds to produce more aromatics [64]. Maximum aromatic yield (≈25 wt.%) was obtained using ZSM-5 with the highest acidity (SiO_2_/Al_2_O_3_ = 30), while the lowest yield (≈7.99 wt.%) was obtained with the least acidic catalyst (SiO_2_/Al_2_O_3_ = 280).

Additionally, doping of other metal elements such as Mn, Co, Ni, Ga, Ce or Pt into ZSM-5 based catalysts can be efficient to regulate the strength and density of acidic sites in zeolite. For instance, in a study by Vichapund et al., it was reported that catalytic pyrolysis of jatropha residues (cellulose: 59.2%, hemicellulose: 18%, lignin: 22.8%) with 3 wt.% Ni-ZSM-5 catalyst provided an ideal environment for the oligomerization, cyclization and dehydrogenation of small olefins, all of which improved the yield of aromatic compounds [65]. In another study, Zhou et al. used silicon carbide foam supported on ZSM-5 catalyst for the microwave-assisted pyrolysis of biomass and reported that increasing the catalyst-to-biomass ratio (0 to ½) improved the quality of bio-oil at the cost of its yield (yield reduced from 40.2% to 33.5%) [66]. The lower bio-oil yield with higher gas yield is attributed to the enhanced catalytic conversion by ZSM-5 catalyst; while primary pyrolytic vapor enters the pore of ZSM-5, oxygen is removed in the forms of CO, CO_2_, and H_2_O through decarbonylation, decarboxylation and dehydration reactions. It was also reported that the catalyst was capable of retaining its activity up to a catalyst-to-biomass ratio of 1/10, which outperformed ZSM-5 catalyst configurations reported in other studies while also allowing its regeneration for reuse with preserved material and catalytic properties for up to seven reaction–regeneration cycles. In another study by Jorge et al. [67], a series of new catalysts consisting of magnetically recoverable β-zeolites exchanged with transition metals showed significant potential for catalytic valorization of hemicellulosic biomass compounds. In particular, Pd-exchanged catalyst showed enhanced efficiency for conversion of both furfural and furfuryl alcohol to isopropyl levulinate.

## 3. Role of Nanotechnology in Bioenergy Production

### 3.1. Enzyme Immobilization

Enzyme immobilization on support materials is useful to enhance enzyme characteristics such as high activity at most pH values, reusability and selectivity and reduce their inhibition. Some studies have reported that immobilization of cellulase enzyme makes it more resistant to structural alterations which may be caused by increase in the temperature [29]. An excellent enzyme immobilization on solid support ensures good distribution of the catalyst with minimal agglomeration. For instance, covalent binding between the support and enzyme have been reported to increase the enzyme activity [53,69]. Other than covalent binding, approaches such as adsorption, ionic bonding, entrapment and encapsulation have been reported for enzyme immobilization. In comparison to other NPs, MNPs have gained significant interest as promising support carriers in enzyme immobilization due to factors highlighted in Section 2.1 of this work. Most importantly, in enzyme immobilization, the large surface-area-to-volume ratio of MNPs is highly advantageous to loading a large number of enzymes on their surface, which in turn increases the catalytic activity of the enzymes. Several techniques can be used for immobilization of enzymes on the MNP support. For example, MNP functionalization for cellulase immobilization can be on silica-functionalized MNP, amino-functionalized MNPs, composite-functionalized MNPs, chitosan-functionalized MNPs or carrier-free functionalized MNPs.

For instance, sulfonated magnetic carbonaceous nanoparticles, which were used for the hydrolysis of various lignocellulosic biomass, showed significant levels of glucose yields of jatropha, bagasse and plukenetia hulls of 35.6%, 58.3% and 35.8%, respectively, with the capability of recycling the catalyst at least seven times with a high catalyst recovery rate of 92.8% [70]. In another study by Goh et al., enzyme immobilization was performed on magnetic single-walled carbon nanotubes for application in biofuel production. It was reported that the enzyme was recyclable, which reduced the cost of biofuel production while also retaining their activity for a month in acetate buffer at 4°C [71]. In another study, 93% binding efficiency and 50% activity retainment after 16th cycle was achieved with a nanobiocatalyst system for biofuel production using functionalized magnetic nanoparticles immobilized with β-glucosidase isolated from fungus [72]. In biodiesel production, immobilization of lipase on polydopamine-coated magnetic nanoparticles enhanced the pH and thermal stability while achieving 73.9% binding efficiency, more than 70% of activity even after 21 repeated cycles and easy recoverability from reaction mixture [73]. Similar findings were also reported in a study by Hu et al., where immobilization on magnetic nanoparticles increased biodiesel production by 95% when a reaction was performed at 65 °C for a duration of 3 h. Additionally, the catalyst was reusable 14 times while allowing recoverability of more than 90% [74]. To date, various MNPs have been explored as support carriers in enzyme immobilization. A summary of the various MNPS that have been used as the support carriers in enzyme immobilization and their effect on bioenergy production is summarized in Table 1.

### 3.2. Biogas Production

In the biogas production process, nanoparticles have proven to show promising results in the anaerobic processes (biochemical processes to convert various organic materials into biogas and its constituents), in particular as electron donors, acceptors and cofactors of key enzymes such as [Fe]- and [Ni-Fe]-hydrogenase [84]. For example, nanoparticles increase the hydrolysis of organic matter due to their large surface-area-to-volume ratio, which enables microorganisms to bind onto the active sites of the molecule. This subsequently stimulates the biochemical process for the activity of hydrogenase enzymes and ferredoxins. Various nanoparticles have been used in the anaerobic digestion process such as zero-valent metals, metal oxides and carbon-based nanomaterials.

Generally, the anaerobic digestion process involves four important steps: (i) hydrolysis, (ii) acidogenesis, (iii) acetogenesis and (iv) methanogenesis. Nanoparticles such as carbon-based materials have been reported to be useful in all the four stages. For example, in a study by Velimirovic et al. [85], carbon-based nanomaterials in the hydrolysis stage, when available as electron donors, can lower the oxygen reduction potential of the water environment, which is highly beneficial in the hydrolysis reaction. In the acidogenesis and acetogenesis processes, studies have reported that the addition of 12.5 g/L biochar enhanced the biohydrogen production from 22.6 to 96.3 mL/g [86], the addition of 8.3 g/L of biochar increased the biohydrogen yield from 750.4 to 944.5 mL/L from food waste [87], and the addition of 600 mg/L of sawdust biochar improved biohydrogen production from 31.5 to 36.5 mL [88]. In all the aforementioned studies, it was highlighted that addition of carbon-based nanomaterials enhanced the performance of acid formation processes, which resulted in improved yield of hydrogen. Similar enhancement in biogas production were also reported in other studies using zero-valent ions (ZVI); i.e., 0.1 wt.% ZVI using waste-activated sludge enhanced biogas production by 30.4% [89], whereas the highest methane formation rate (0.310 mmol CH_4_ formed/mol Fe^0^. day) was reported using the finest grade iron ZVI [90].

Studies have also been performed to study the effect of nanoparticles on microbial communities in the anaerobic digestion process. For example, Wang et al. used four types of nanoparticles, i.e., ZVI, Ag, Fe_2_O_3_ and MgO, to investigate their effect on biogas production from waste-activated sludge, and it was reported that ZVI (10 mg/g) and Fe_2_O_3_ (100 mg/g TSS) increased biogas production by 120% and 117%, respectively, which suggests that ZVI and Fe_2_O_3_ nanoparticles improve the activity of the methanogenic bacteria [91]. This was also supported in another study by Yang et al., which reported that addition of ZVI nanoparticles increases the population of methanogens in an anaerobic digester [92].

### 3.3. Biohydrogen Production

Biohydrogen production is performed by anaerobic bacteria via metabolic routes to generate molecular hydrogen. It has been reported that the activity of these microorganisms can be enhanced with the use of nanoparticles to increase electron transfers and kinetics in metabolic processes to produce biohydrogen due to their capability to react faster with electron donors. The role of nanoparticles in various biohydrogen process is as described below.

#### 3.3.1. Dark Fermentative Biohydrogen Process

Nanoparticles have been reported to improve the dark fermentation process. For instance, 5 nm gold nanoparticles improved substrate utilization and biohydrogen yield by 56% and 46%, respectively, owing to the large surface-area-to-volume ratio of gold nanoparticles, which provided a stimulatory effect to produce biohydrogen [93]. It has also been highlighted in other studies that gold nanoparticles enhance the activity of biohydrogen-producing enzymes, which mediate the transfer of electrons such as [Fe-Fe]- and [Ni-Fe]-hydrogenases and ferredoxins [94,95]. In other studies, zero-valent iron (Fe^0^) nanoparticles were used for dark fermentation of grass, and it was shown that Fe^0^ nanoparticles stimulated activity of a hydrogenase enzyme to produce a higher yield of biohydrogen (i.e., maximum hydrogen yield of 64.7 mL/g dry grass; 73% higher than control experiments) [96]. Other studies have also reported the use of metallic nanoparticles such as Pb, Ag and Cu along with FeO nanoparticles immobilized on porous silica (SiO_2_), and highest yield and production rate was achieved using FeO nanoparticles, i.e., 38% and 58%, respectively, in comparison to control experiments [97]. Ni-graphene-based nanoparticles have also been reported to enhance dark fermentative biohydrogen production, in a study where maximum yield of 41.3 H_2_/g COD with 105% increase in H_2_ yield was obtained [98].

#### 3.3.2. Photo-Fermentative Biohydrogen Production

Nanoparticles have also drawn significant interest as means to improve the biomass growth, photosynthetic activity, nitrogen metabolism and protein level of microalgal species to produce biohydrogen. The key roles of nanoparticles in photo-fermentative biohydrogen production are as summarized below:

1. Nanoparticles behave as the catalytic agents to generate metabolic pathways to promote synthesis of chlorophyll a, chlorophyll b, carotenoids and anthocyanin, lipid production and nitrogen metabolism [99,100].
2. Nanoparticles enhance production of carbohydrates, which results in increased growth of algal cells. For example, silica nanoparticles enhanced growth of microalgal cells (measured from chlorophyll concentration), because silica nanoparticles scattered light within a reactor to ensure uniform light distribution during the photosynthetic process, which in turn promoted growth of microalgal cells [101]. Similar findings were also reported using zero-valent iron (Fe^0^) [102] and TiO_2_ nanoparticles, which increased chlorophyll and carotenoid pigments [103].
3. Nanoparticles enhance activity of key enzymes for metabolism of microalgal species such as glutamate dehydrogenase, glutamate–pyruvate transaminase, glutamine synthase and nitrate reductase [104,105]. The capability of nanoparticles to maintain the pH of a medium and to promote the activity of hydrogenase enzymes and substrate hydrolysis may promote higher biohydrogen yield by enhancing biohydrogen-producing metabolic pathways such as acetate and butyrate reactions [32,103,106].

#### 3.3.3. Photocatalytic Hydrogen Production

Photocatalytic biohydrogen production involves the breaking of water molecules into H_2_ and O_2_ in the presence of an illuminating source. A photocatalyst such as TiO_2_ has been commonly preferred owing to its high photocatalytic performance, non-toxicity, chemical stability and cost effectiveness [107,108]. For instance, nanoparticles such as Pt-TiO_2_-activated carbon generated a high hydrogen production rate of 7490 µmol/h/g photocatalyst, which is 75 times higher than conventional Aeroxide TiO_2_ P25 catalyst [107]. Other studies have reported the combination of TiO_2_-graphene has higher light absorption efficiency than TiO_2_ alone, which resulted in higher charge separation efficiency to yield higher hydrogen [109]. The effect of various nanoparticles on the hydrogen yield rate is summarized in Table 2.

### 3.4. Bioethanol Production

**Figure 6:**
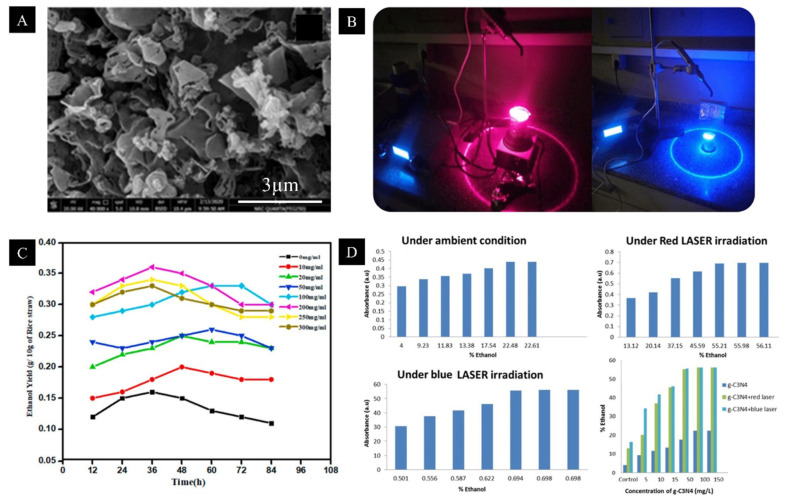
(**A**) SEM image of prepared g-C_3_N_4_ nanoparticles used to increase bioethanol production. (**B**) Irradiation using blue and red laser to increase bioethanol production from potato waste. (**C**) Effect of ZnO nanoparticles on bioethanol yield from rice straw. (**D**) Effect of various processing conditions of g-C_3_N_4_ nanoparticles on the production of bioethanol. Reproduced with permission from [124] Springer Nature and [125] Elsevier.

Generally, lignocellulosic materials are processed for bioethanol production via three major operations, which include (i) pretreatment for delignification to liberate the cellulose and hemicellulose, (ii) hydrolysis to produce fermentable sugars such as glucose, xylose, arabinose, galactose or mannose and (iii) fermentation of reducing sugars. In a very recent study, Saeed et al. [124] used graphitic carbon nitride (g-C_3_N_4_) nanomaterials (Figure 6A) and laser irradiation (Figure 6B) to increase bioethanol production from potato waste. Authors reported that the control sample without laser irradiation or g-C_3_N_4_ showed only 4% yield of bioethanol, while the addition of g-C_3_N_4_ coupled with laser irradiation increased bioethanol yield to 56.8% (Figure 6D).

In another study by Gupta et al. [125], zinc oxide nanoparticles were used to enhance bioethanol production from rice straw, and a maximum ethanol yield of 0.0359 g/g dry weight-based plant biomass was attained at 200 mg/L concentration of ZnO nanoparticles (Figure 6C). Additionally, the possibility for reusability and recovery of the nanoparticles makes the entire process more economical. Similarly, in another study by Ivanova et al. [126], it was reported that bioethanol fermentation was enhanced with the use of alginate magnetic nanoparticles entrapped with yeast cells, with the productivity rate reaching up to 264 g/Lh at 70% particle loading. It was also interesting to note that the magnetic particles with fixed yeast cells were stable for more than a month at 4 °C in saline condition. In a similar vein, Kim et al. [127] reported enhanced bioethanol production in syngas fermentation using methyl-functionalized silica nanoparticles. After 9 h of cultivation time, it was reported that ethanol concentrations without and with nanoparticles were 0.1150 and 0.3060 g/L,w respectively, which indicates that ethanol production was enhanced by 166.1% by the use of nanoparticles. When producing liquid fuels through fermentation, it is expected that nanomaterials will influence the biochemical conversion process by affecting the enzymatic activity or the mass transfer rate.

## 4. Concluding Remarks and Future Prospects

Development and usage of bioenergy is of great importance towards a greener future. Performance enhancement such as enhancement of catalytic and microbe activities, and reduction in inhibition formation have been shown to result from using nanomaterials. For example, nanoparticles have been used as additives to improve the working performance of biodiesel [128].

Generally, these nanomaterials offer large surface area, highly modifiable framework, and high tunability in their performance. All of them have shown good recoverability after several runs. MNPs can be coated and immobilized as catalyst during hydrolysis, as well as possessing high recoverability. Coated MNPs are more resistant against chemical destruction during synthesis stages. Enzyme immobilization is highly compatible with the structural arrangement of MWCNTs, as well as mesoporosity in metal oxides and silica, further enhancing the catalytic activities. Biocompatibility of silica is advantageous in enzyme immobilization, whereas the silanol group on silica can help to control the surface hydrophobicity and enhance catalytic performance such as durability, activity and selectivity in acid-catalyzed chemical reactions. MOF is employed as a heterogenous catalyst owing to its high porosity and ordered structure, enabling more substrate transfer. Zeolites usage in aqueous phase is limited as they are sensitive to hot water.

Studies on employing nanotechnology in bioenergy generation have been conducted since the late 2000s. The incorporation of nanotechnology can be highly advantageous, in particular when used to design catalysts at the atomic level to increase selectivity, efficiency, operational temperature range, and many important properties of the catalyst. However, despite several advantages of employing nanotechnology in such a field, further understanding its interaction and relevant mechanism is required, as nanomaterials exhibit their own characteristics when interacting with biomass. For example, enzyme immobilization, improved catalytic activity and biohydrogen production are the important results when employing nanomaterials for bioenergy generation purposes. Different nanomaterials have been reviewed in this paper, and the relevant interactions are outlined.

Enzyme immobilization can be considered as the main role of nanomaterials in terms of bioenergy production owing to the large surface-area-to-volume ratio of nanoparticles which increases the enzyme activity at the same time. Enhancement of enzyme activity at most pH values, reusability, selectivity and inhibition reduction are the key criteria in enzyme immobilization. Nanomaterials acting as support carriers are successful in obtaining improved catalytic activity and stability. In terms of biogas production, microorganisms are able to bind to the active site of nanoparticles, increasing the organic matter hydrolysis rate and thereby improving the anaerobic digestion process. Carbon-based nanomaterials have been shown to improve all four important steps in the anaerobic digestion process. Additionally, the nanoparticles aid the population increase in methanogens in an anaerobic digester. For biohydrogen production, nanoparticles catalyze the metabolic pathway for chlorophyll synthesis, as well as carbohydrates.

Despite the advantages offered by nanomaterials for bioenergy generation, there are still several issues which hinders their widescale usage. From an economic perspective, nanomaterials are relatively expensive, which inhibits widescale usage of nanomaterials for industrial scale. However, carbon-based nanomaterials are becoming cheaper currently, owing to the efforts from various industrial producers. Considerable efforts are still required for further reduction in cost of other nanomaterials. Besides, recoverability issue of nanomaterials leads to loss of profit. With further research, the recoverability issue can be resolved in the near future. Additionally, although toxicity induced by nanoparticles has hitherto not been understood, metal nanocatalyst or nanoparticles emitted as exhaust from vehicles or industries may be deposited in lung tissue via respiration, disrupt normal functioning of human cells or cause respiratory ailments such as asthma or bronchitis. Thus, safety assessment is of utmost importance to minimize such concerns when utilizing nanomaterials for bioenergy generation.

## Figures and Tables

**Table 1:** Summary of various MNPs explored as support carriers in enzyme immobilization for bioenergy generation.

Bioenergy	MNPs	Feedstock	Enzyme	Summary	Ref.
Bioethanol	Fe_3_O_4_	*Aspergillus fumigatus* AA001	Cellulase	Thermal stability for 8 h at 70 °C	[18]
	Fe_3_O_4_	Potato peels	Amylase/Amyloglucosidase	Bioethanol yield: 93%	[75]
	Fe_3_O_4_	*Allamanda schottii* L.	Cellulase	Bioethanol yield (free enzymes): 182 g/L; (immobilized enzymes): 252 g/L	[76]
	FeCl_3_	*Sesbania aculeata*	Cellulase	Bioethanol yield: 5.31 g/L	[29]
	MnO_2_	*Aspergillus fumigatus* JCF	Cellulase	Cellulase binding efficiency of 75% Bioethanol yield: 21.96 g/L	[77]
Biodiesel	Fe_2_O_3_	*Neochloris oleoabundans*	Lipase	Maximum biodiesel yield: 81%	[78]
	Fe_3_O_4_	Soybean oil	Lipase	Biodiesel yield: 90% with 60% immobilized lipase	[79]
	Fe_3_O_4_	Soybean oil	Lipase	Conversion rate: 47.60% after 24 h; >30% enzyme activity even after 10 cycles	[30]
	Fe_3_O_4_	Soybean oil	Lipase	Conversion rate: 88% after 192 h and 75% after 240 h	[80]
	Fe_3_O_4_	Microalgae (*Chlorella vulgaris*)	Lipase	Maximum biodiesel yield: 97.1%	[81]
	Fe_3_O_4_	Jatropha curcas oil	Rhizomucor miehei lipase	Maximum biodiesel yield: 70%	[82]
	Polyporous magnetic cellulose beads (PMCBs)	*Yellow horn* seed oil	Candida antarctica lipase B	Maximum biodiesel yield: 92.3%; catalyst easily removed by magnet and can be recycled at least 5 times.	[83]

**Table 2:** Effect of various nanoparticles on the H_2_ yield rate. Modified from [110].

NP	Feedstock	Microorganism	H_2_ Yield Rate	H_2_ Yield Increase (%)	Ref.
Au	Artificial wastewater	*Clostridium butyricum*	4.48 mol H_2_/mol sucrose	61.7	[93]
Ag	Inorganic salts	*Clostridium butyricum*	2.48 mol H_2_/mol glucose	67.5	[111]
Pd	Glucose	*Enterobacter cloacae* + mixed culture	2.48 mol H_2_/mol glucose	6.4	[112]
Ni	Inorganic salts	Granular sludge	2.54 mol H_2_/mol glucose	22.7	[113]
Cu	Glucose	*Clostridium acetobutylicum*	1.74 mol H_2_/mol glucose	N/A	[114]
Cu	Glucose	*Enterobacter clocae*	1.44 mol H_2_/mol glucose	N/A	[114]
Fe	Inorganic salts	*Enterobacter clocae*	1.9 mol H_2_/mol glucose	68.4	[115]
Fe	Growth medium	*Rhodobacter sphaeroides + Escherichia coli*	3.1 mol H_2_/mol malate	N/A	[116]
Fe_2_O_3_	Casava starch	*Enterobacter aerogenes*	192.4 mL H_2_/g casava starch	17	[117]
Fe_2_O_3_	Distillery wastewater	*Mixed culture*	44.28 mL H_2_/g COD	N/A	[118]
Fe_2_O_3_	Growth medium	*Clostridium acetobutylicum*	2.33 mol H_2_/mol glucose	52	[119]
FeO	Growth medium	Mixed culture	1.92 mol H_2_/mol glucose	7.9	[120]
TiO_2_	Growth medium	*Rhodopseudomonas palustris*	N/A	46.1	[121]
SiO_2_	Air: CO_2_ (97:3)	*Chlamydomonas reinhardtii* CC124	3121.5 H_2_/L/h	45.2	[101]
Biochar	Municipal solid waste	*Enterobacter aerogenes* + *Escherichia coli*	96.3 mL/g	N/A	[86]
Biochar	Food waste	N/A	944.5 mL/L	31	[87]
Biochar + ZV iron NP	Grass biomass	N/A	50.6 mL/g dry grass	89.8	[88]
Ni + graphene	Industrial wastewater	Mixed culture	41.3 mL/gCOD	105	[98]
CNT	Glucose	Anaerobic sludge	2.45 mol/mol substrate	N/A	[122]
Hematite + TiO_2_	Glucose	*Clostridium pasteurianum* CH5	2.20 mol/mol substrate	5	[123]
